# Matched-pair analysis of mCRPC patients receiving ^177^Lu-labeled PSMA-targeted radioligand therapy in a 4-week versus 6-week treatment interval

**DOI:** 10.1186/s13550-024-01143-0

**Published:** 2024-10-14

**Authors:** Amir Karimzadeh, Charlotte-Sophie Hecker, Matthias M. Heck, Robert Tauber, Calogero D’Alessandria, Wolfgang A. Weber, Matthias Eiber, Isabel Rauscher

**Affiliations:** 1 Department of Nuclear Medicine, School of Medicine, Technical University of Munich, Munich, Germany; 2 Department of Diagnostic and Interventional Radiology and Nuclear Medicine, University Medical Center Hamburg-Eppendorf, Martinistr. 52, 20246 Hamburg, Germany; 3 Department of Urology, School of Medicine, Technical University of Munich, Munich, Germany

**Keywords:** Prostate-specific membrane antigen (PSMA), Radioligand therapy (RLT), Metastatic castration-resistant prostate cancer (mCRPC), 4-week treatment interval

## Abstract

**Background:**

The optimal regimen for ^177^Lu-labeled prostate-specific membrane antigen-targeted radioligand therapy, including treatment intervals, remains under study, with evidence suggesting shorter intervals could benefit patients with high disease volume and rapid progression. This retrospective analysis evaluated treatment toxicity, PSA response, PSA-progression-free survival (PSA-PFS), and overall survival (OS) in matched cohorts of mCRPC patients receiving 177Lu-PSMA-RLT at 4-week versus 6-week intervals.

**Results:**

A PSA response (PSA decline ≥ 50%) was achieved in 47.8% and 21.7% of patients in the 4-week and 6-week treatment interval groups, respectively (*p* = 0.12). There was a trend towards longer PSA-PFS in the 4-week group compared to the 6-week group (median PSA-PFS, 26.0 weeks vs. 18.0 weeks; HR 0.6; *p* = 0.2). Although not statistically significant, there was a trend towards shorter OS in the 4-week group compared to the 6-week group (median OS, 15.1 months vs. 18.4 months; HR 1.3; *p* = 0.5). The 4-week group had a significantly greater decrease in leucocyte and platelet counts compared to the 6-week group (38.5% vs. 18.2% and 26.7% vs. 10.7%; *p* = 0.047 and *p* = 0.02). Severe adverse events were modest in both groups.

**Conclusions:**

Intensifying treatment intervals from 6 weeks to 4 weeks showed some improvements in PSA response and PSA-PFS for mCRPC patients, but did not significantly affect OS. Additionally, bone marrow reserve was significantly reduced with the intensified regimen. Therefore, the overall benefit remains uncertain, and further prospective studies are needed to compare 4-week and 6-week intervals regarding toxicity, treatment response, and outcome.

## Introduction

^177^Lu-labeled prostate-specific membrane antigen (PSMA)-targeted radioligand therapy (RLT) has become an established treatment option for patients with metastatic castration-resistant prostate cancer (mCRPC). The efficacy and low toxicity of ^177^Lu-labeled PSMA-RLT have been documented initially by retrospective analyses followed by prospective phase II and III trials resulting in its approval by various regulatory bodies worldwide [1–6].

Since its introduction, the optimal treatment regimen for ^177^Lu-labeled PSMA-RLT has remained a subject of research, focusing on finding the right treatment activity and ideal treatment intervals. Initial retrospective analyses explored treatment intervals ranging from six to twelve weeks. In this context, the impact of a higher 7.5 GBq activity every 6 weeks versus a lower 6 GBq activity every 8 weeks was analyzed [7]. Results from this analysis showed a trend towards better response rates and longer survival times with the higher dose and shorter intervals, without compromising safety. Although not statistically significant, these differences suggested that shorter treatment intervals could potentially enhance treatment response and outcome of ^177^Lu-labeled PSMA-RLT. Subsequently, the prospective phase III VISION trial, established a six-week treatment interval with a fixed activity of 7.4 GBq [5]. However, almost one third of the trial patients did not respond to this treatment regimen with one potential hypothesis that this fixed scheme is insufficient for a subset of mCRPC patients. Given the favorable safety profile of ^177^Lu-labeled PSMA-RLT, recent analyses have explored even shorter intervals of four weeks, demonstrating promising results with low toxicity and treatment responses [8, 9]. These findings suggest that shorter intervals between treatment regimens might be a reasonable approach particularly for patients with high disease volume and rapid disease progression. Despite these promising findings, there is still a lack of comparative data between four- and six-week ^177^Lu-labeled PSMA-RLT regimens. Therefore, the aim of this retrospective analysis was to evaluate treatment toxicity, prostate specific antigen (PSA) response, PSA-progression-free survival (PFS), and overall survival (OS) using matched-pair cohorts of patients receiving ^177^Lu-labeled PSMA-RLT in 4-week versus 6-week treatment intervals.

## Materials and methods

### Patients and [^177^Lu]Lu-PSMA-I&T

This retrospective analysis included 46 mCRPC patients who received a standard activity of approximately 7.4 GBq [^177^Lu]Lu-PSMA-I&T between December 2014 and February 2022. The treatment activity could be slightly adopted based on e.g. lab tests and tumor burden. Before treatment, sufficient PSMA expression was confirmed by PSMA-ligand positron emission tomography (PET). Only patients with PSMA-ligand uptake in tumor lesions at least as high as liver background uptake were treated. 23 patients treated on a 4-week interval based on the decision by the treating nuclear medicine physician (e.g. due to critical treatment pressure) were matched with an equal number of patients receiving treatment on a standard 6-week interval. Matching criteria included Gleason score (≤ 6 versus 7a/b versus ≥ 8), time interval between initial diagnosis and 1st cycle of [^177^Lu]Lu-PSMA-I&T, baseline lactate dehydrogenase (LDH) levels, previous second generation antihormonal treatment (abiraterone and/or enzalutamide or none of them), previous taxane-based chemotherapy (docetaxel or cabazitaxel or docetaxel and cabazitaxel or none of them in case of ineligibility) and molecular imaging TNM classification obtained from baseline PSMA-ligand PET/CT scans with exact matches for M1a, M1b and overall M1c. [^177^Lu]Lu-PSMA-I&T was prepared according to good manufacturing practice and the German Medicinal Products Act (AMG § 13 2b). All patients gave written informed consent. The institutional ethics committee approved this retrospective analysis under the reference number 115/18S. Patients were treated under the conditions of the Declaration of Helsinki article 37 „unproven interventions in clinical practice “.

### Image analysis

All patients gave written informed consent prior to imaging with PSMA-ligand PET/CT. PSMA-ligands ([^68^Ga]Ga-PSMA-11, [^18^F]PSMA-1007, [^18^F]rhPSMA-7 or [^18^F]rhPSMA-7.3) were administered via intravenous bolus, with PET acquisition starting approximately 60–90 min post-injection. Patients were given a diluted oral contrast medium (300 mg of Telebrix; Guerbet) and 10 mg of furosemide. PET/CT imaging was performed using either a Biograph mCT Flow scanner or a Biograph Vision scanner (both from Siemens Medical Solutions). Scans were conducted in 3-dimensional mode with acquisition times of 0.8 mm/s (mCT Flow) and 1.1 mm/s (Vision), respectively. PET images were reconstructed using ordered-subset expectation maximization (TrueX, 4 iterations, 8 subsets) and then smoothed with a Gaussian filter (3 mm full width at half maximum). A diagnostic CT scan was first performed in the portal venous phase 80 s after intravenous injection of an iodinated contrast agent (Imeron 300; Bracco Imaging), followed by the PET scan.

### Toxicity, response and outcome assessment

**Fig. 1:**
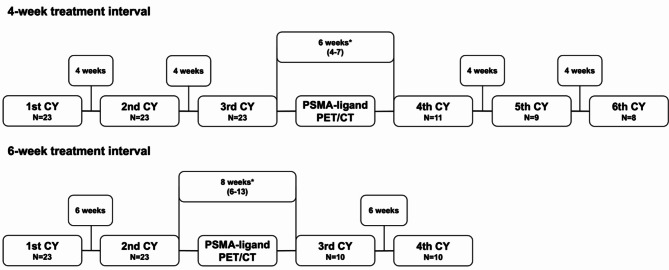
Treatment scheme in the 4-week and 6-week interval groups. Asterisks indicate deviations from the 4-week and 6-week treatment intervals, given as median (range), due to individual delays in conducting the first interim PSMA-ligand PET/CT

The following pre-therapeutic parameters were collected: age, alanine aminotransferase, alkaline phosphatase, aspartate aminotransferase, creatinine, Gleason score, LDH, leucocytes, haemoglobin, platelets and PSA. Toxicity was assessed by comparing baseline parameters with those at the last available cycle. Changes in blood cell counts and creatinine levels were graded according to the Common Terminology Criteria for Adverse Events version 5.0 (CTCAE v. 5.0) [10]. The best PSA response, defined as the greatest decrease or smallest increase in PSA from baseline, was also evaluated. According to Prostate Cancer Clinical Trials Working Group 3, PSA progression was defined as PSA increase ≥ 25% and ≥ 2 ng/ml above the nadir after initial PSA decline or PSA increase ≥ 25% and ≥ 2 ng/ml from baseline in case with no PSA decline [11]. Toxicity, PSA response, and PSA progression were assessed up to the second interim PSMA-ligand PET/CT to ensure that only the period during which patients received their treatment in the designated 4- or 6-week intervals was captured. This cutoff was necessary because after the second interim PSMA-ligand PET/CT some patients transitioned from a 4-week to a 6-week treatment schedule based on the nuclear medicine physician’s decision, which was made in response to sufficient clinical, imaging and/or biochemical improvements and to minimize the risk of further hematotoxic deterioration. In cases without PSA progression, patients were censored at the time of the second interim PSMA-ligand PET/CT to ensure that PSA progression was only captured during the period when patients were receiving therapy in the designated 4- or 6-week intervals. Among those on the 4-week schedule, 10 had a gap longer than 4 weeks between their 3rd and 4th treatment cycle. Similarly, in the 6-week schedule group, 7 had a gap longer than 6 weeks between their 2nd and 3rd treatment cylces (Fig. 1). This deviation was due to individual delays in conducting the first interim PSMA-ligand PET/CT.

### Statistical analysis

Descriptive data is presented as median and range in parentheses. Comparisons between the two groups, as well as within each group were performed using the Wilcoxon signed-rank test. For PSA-PFS and OS, Kaplan-Meier method was used for estimation of event time distribution and logrank test was used for group comparisons. For comparison of dichotomous variables, Fisher’s exact test was applied. The corresponding hazard ratios (HR) and 95% confidence intervals (CI) are presented. A p value of < 0.05 was considered statistically significant. For statistical analyses GraphPad Prism version 10.1.0 (264) for MAC (GraphPad Software, San Diego, California USA, www.graphpad.com) was used.

## Results

### Patients’ characteristics

Patients´ characteristics are presented in Table 1. The median time between initial diagnosis and ^177^Lu-labeled PSMA-RLT initiation was 4 years and 4.5 years in patients with 4-week and 6-week treatment interval, respectively. The median Gleason score was 9 in both cohorts (*n* = 4 with Gleason score ≤ 6, *n* = 6 with Gleason Score 7a/b and *n* = 36 with Gleason score ≥ 8). For baseline LDH levels, no significant differences between the groups were observed (4-week: 263 ng/ml (171–1355) vs. 6-week: 264 ng/ml (188–773); *p* = 0.99). Except for baseline PSA (4-week: 144.0 ng/ml (3.7–2760.0) vs. 6-week: 60.8 ng/ml (6.2–857); *p* = 0.006), serum chemistry and blood counts did not differ between both groups. 18 and 16 patients in the 4-week treatment interval group and 20 and 14 patients in the 6-week treatment interval group (*n* = 17 with abiraterone and/or enzalutamide in both groups) received pretreatment with abiraterone and enzalutamide, respectively. Pretreatment with docetaxel and cabazitaxel was assessed in 17 (73.9%) and 4 (17.4%) patients. According to the molecular imaging TNM classification, 15 (65.2%), 23 (100%) and 5 (21.7%) patients presented with extrapelvic lymph node metastases (M1a), bone metastases (M1b) and visceral metastase (M1c) status at baseline, respectively. Up to the second interim PSMA-ligand PET/CT, patients in the 4-week treatment interval group received a total of 97 cycles (3 to 6 cycles per patient) of [^177^Lu]Lu-PSMA-I&T with a median cumulative activity of 23.6 GBq (21.3–46.2) per patient. Patients in the 6-week treatment interval group received a total of 66 cycles (2 to 4 cycles per patient) with a median cumulative activity of 15.8 GBq (13.6–33.4) per patient. Median time on treatment up to the second interim PSMA-ligand PET/CT was 12.3 and 10.9 weeks in the 4-week and 6-week treatment interval group, respectively. Differences in number of applied treatment cycles per patient (*p* = 0.0002) and cumulative activity per patient (*p* < 0.0001) were significant.

**Table 1:** Patient characteristics

Clinical variables	4 week treatment interval	6 week treatment interval	*P*-value
Age at first cycle of PSMA RLT (years), median (range)	67 (52–83)	70 (58–87)	0.15
Time period between initial diagnosis and 1st RLT (years), median (range)#	4 (1–16)	4.5 (2–25)	0.2
No. of treatment cycles up to the second Interim PSMA-ligand PET imaging	97	66	**0.0002**
Cumulative activity per patient up to the second Interim PSMA-ligand PET imaging (GBq), median (range)	23.6 (21.3–46.2)	15.8 (13.6–33.4)	**< 0.0001**
Activity per cycle up to the second Interim PSMA-ligand PET imaging (GBq), median (range)	7.4 (6.9–7.8)	7.5 (3.4–8.4)	0.06
Gleason score, median (range)#	9 (5–9)	9 (6–10)	0.36
**Baseline laboratory values**,** median (range)**			
LDH (U/l)#	263 (171–1355)	264 (188–773)	0.99
Hb (g/dl)	11.5 (8.0-14.9)	11.5 (7.5–14.5)	0.6
Leukocytes (tsd/µl)	6.3 (2.6–13.7)	6.9 (3.5–9.4)	0.87
Platelets (tsd/µl)	224 (124–512)	239 (99–491)	0.58
Creatinine (mg/dl)	0.9 (0.6–1.2)	0.8 (0.7–2.3)	0.82
AST (U/l)	27 (18–115)	28 (14–66)	0.84
ALT (U/l)	16 (7–50)	18 (7–57)	0.81
AP (U/l)	139 (73–759)	117 (41–650)	0.35
PSA (ng/ml)	144.0 (3.7–2760.0)	60.8 (6.2–857)	**0.006**
**Previous systemic treatments**,*** n (%)***			
Abiraterone	18 (78.3)	20 (87.0)	0.7
Enzalutamide	16 (69.6)	14 (60.9)	0.76
Previous second generation antihormonal treatment (Abiraterone and/or Enzalutamide)#	22 (95.7)	22 (95.7)	1.0
Docetaxel#	17 (73.9)	17 (73.9)	1.0
Cabazitaxel#	4 (17.4)	4 (17.4)	1.0
Previous chemotherapy	17 (73.9)	17 (73.9)	1.0
^223^Radium	1 (4.3)	1 (4.3)	1.0
**Site of metastasis**,*** n (%)***			
Lymph node (M1a)#	15 (65.2)	15 (65.2)	1.0
Bone (M1b)#	23 (100)	23 (100)	1.0
Visceral, overall (M1c) #	5 (21.7)	5 (21.7)	1.0
Liver	2 (8.7)	0	
Lung	1 (4.4)	3 (13.0)	
Adrenal	1 (4.4)	2 (8.7)	
Others	2 (8.7)	0	

AP alkaline phosphatase, Hb haemoglobin, LDH lactate dehydrogenase, PSMA RLT prostate-specific membrane antigen targeted radioligand therapy, PSA prostate-specific antigen

# = matching criteria

### Treatment toxicity

**Fig. 2:**
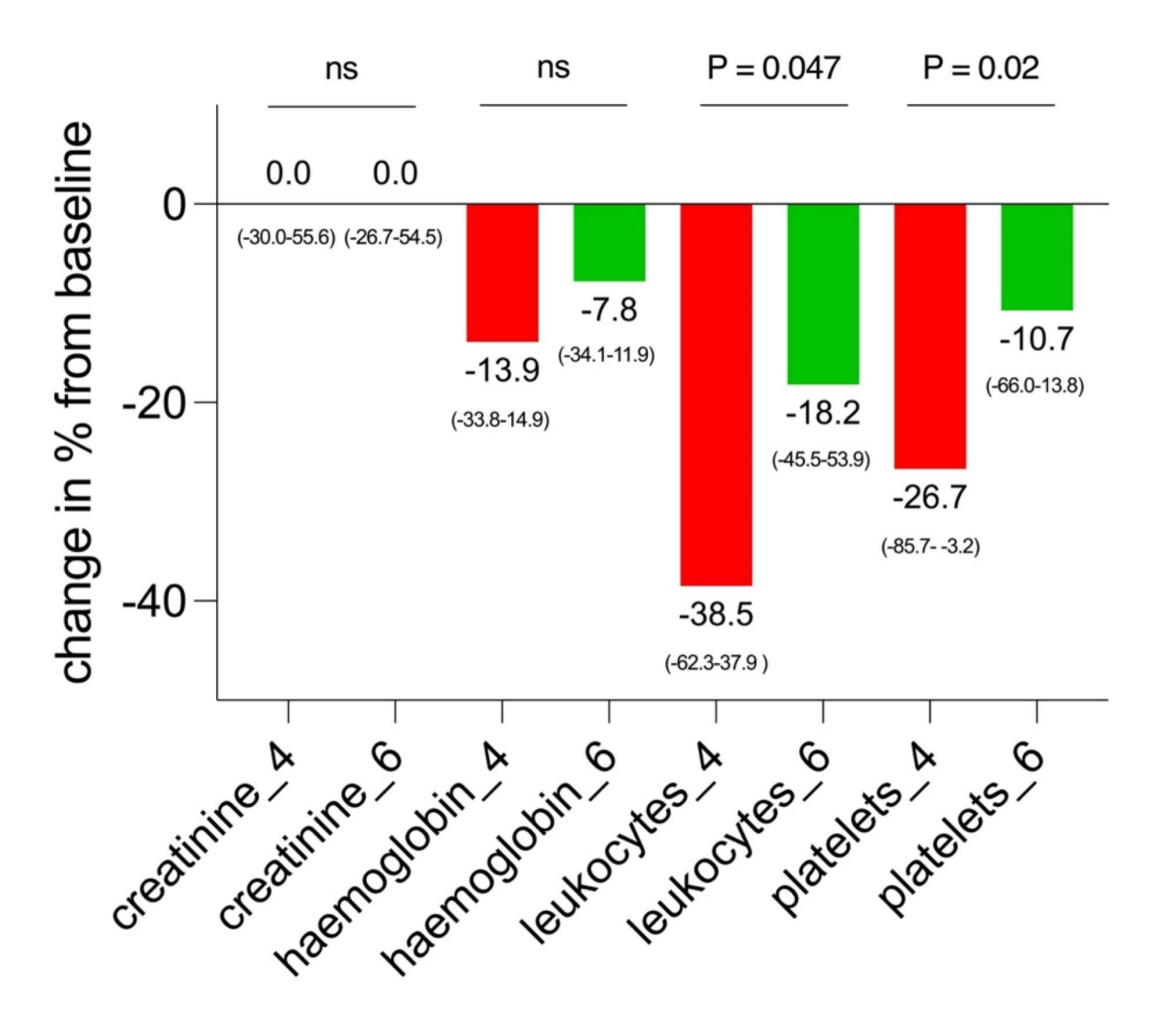
Relative changes in lab values. Creatinine, haemoglobin, leukocyte, and platelet counts at the last available cycle of [^177^Lu]Lu-PSMA-I&T, comparing patients treated every 4 weeks (red) to those treated every 6 weeks (green). A significant *p*-value relative to baseline is indicated as * (< 0.05). Data are represented as median values, with their ranges in brackets

**Fig. 3:**
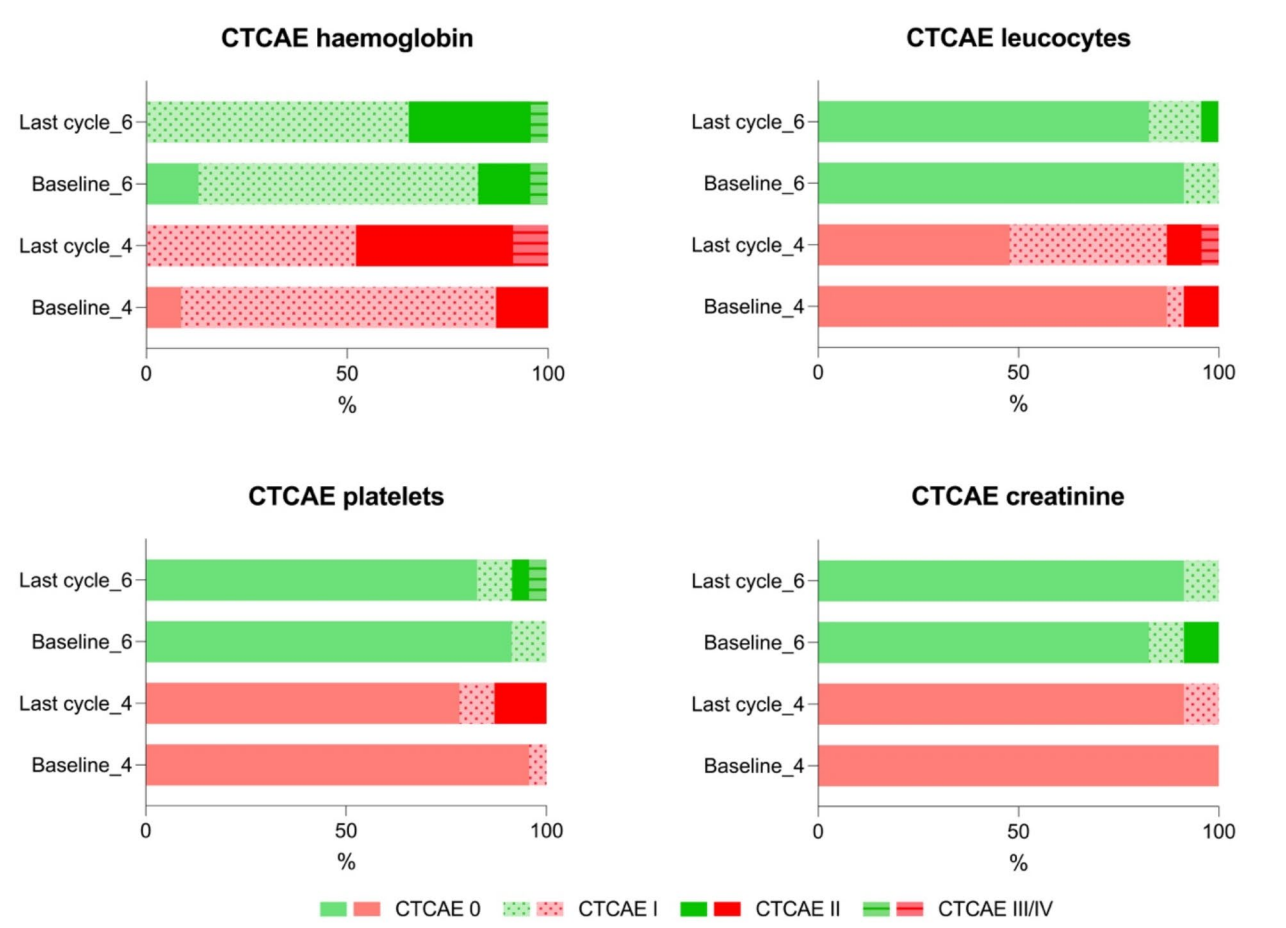
Adverse events based on CTCAE v. 5.0. Adverse events based on CTCAE v. 5.0 for haemoglobin, leukocytes, platelets, and creatinine were compared in percentages between patients undergoing 4-week (red) and 6-week (green) treatment intervals, both at baseline and the final treatment cycle. Grade ≥ III toxicities are indicated by horizontal lines. From baseline, in the 4-week treatment interval group, there were two instances of grade III anemia and one of grade III leukopenia. In the 6-week treatment interval group, one grade III anemia and one grade III thrombocytopenia were observed; however, the same patient already had grade III anemia at baseline. There were no other grade III/IV toxicities in either group

Patients in the 4-week treatment interval group demonstrated a median decrease of haemoglobin, leukocytes and platelets of 13.9%, 38.5%, and 26.7%, respectively (see also Fig. 2). For patients being treated in a 6-week treatment interval, respective declines of 7.8%, 18.2%, and 10.7% were observed. Differences were significant for the decrease of leucocytes (*p* = 0.047) and platelets (*p* = 0.02). There were no relevant changes in creatinine levels between both groups (Table 2). Compared to baseline, two grade III anaemia occurred in patients treated in 4-week intervals (one with grade I and one with grade II anaemia at baseline) and one in the 6-week treatment interval group (already with grade III at baseline) (for details please see Fig. 3). Furthermore, grade III leucopenia was observed in a patient receiving treatment every 4 weeks (at baseline grade II) and one grade III thrombocytopenia occurred in a patient being treated every 6-weeks (at baseline grade II). Apart from that, no further grade III or IV toxicities were observed in both groups.

**Table 2:** Classifications based on CTCAE version 5.0 for haemoglobin, leukocytes, platelets, and creatinine were compared in percentages between patients undergoing 4-week and 6-week treatment intervals, both at baseline and the final treatment cycle. From baseline, in the 4-week treatment interval group, there were two instances of grade III anemia and one of grade III leukopenia. In the 6-week treatment interval group, one grade III anemia and one grade III thrombocytopenia were observed; however, the same patient already had grade III anemia at baseline. There were no other grade III/IV toxicities in either group

		4-week treatment interval					6-week treatment interval				
		Grade 0	Grade 1	Grade 2	Grade 3	Grade 4	Grade 0	Grade 1	Grade 2	Grade 3	Grade 4
Creatinine	Baseline	23/23 (100%)	0	0	0	0	19/23 (82.6%)	2/23 (8.7%)	2/23 (8.7%)	0	0
	Last cycle	21/23 (91.3)	2/23 (8.7%)	0	0	0	21/23 (91.3%)	2/23 (8.7%)	0	0	0
Haemoglobin	Baseline	2/23 (8.7%)	18/23 (78.3)	3/23 (13.0)	0	0	3/23 (13.0%)	16/23 (69.6%)	3/23 (13.0%)	1/23 (4.3%)	0
	Last cycle	0	12/23 (52.2%)	9/23 (39.1%)	2/23 (8.7%)	0	0	15/23 (65.2%)	7/23 (30.4%)	1/23 (4.3%)	0
Platelets	Baseline	22/23 (95.7%)	1/23 (4.3%)	0	0	0	21/23 (91.3%)	0	2/23 (8.7%)	0	0
	Last cycle	18/23 (78.3%)	2/23 (8.7%)	3/23 (13.0%)	0	0	19/23 (82.6%)	1/23 (4.3%)	2/23 (8.7%)	1/23 (4.3%)	0
Leucocytes	Baseline	20/23 (87.0%)	1/23 (4.3%)	2/23 (8.7%)	0	0	21/23 (91.3%)	2/23 (8.7%)	0	0	0
	Last cycle	11/23 (47.8%)	9/23 (39.1%)	2/23 (8.7%)	1/23 (4.3%)	0	19/23 (82.6%)	3/23 (13.0%)	1/23 (4.3%)	0	0

### Treatment response

**Fig. 4:**
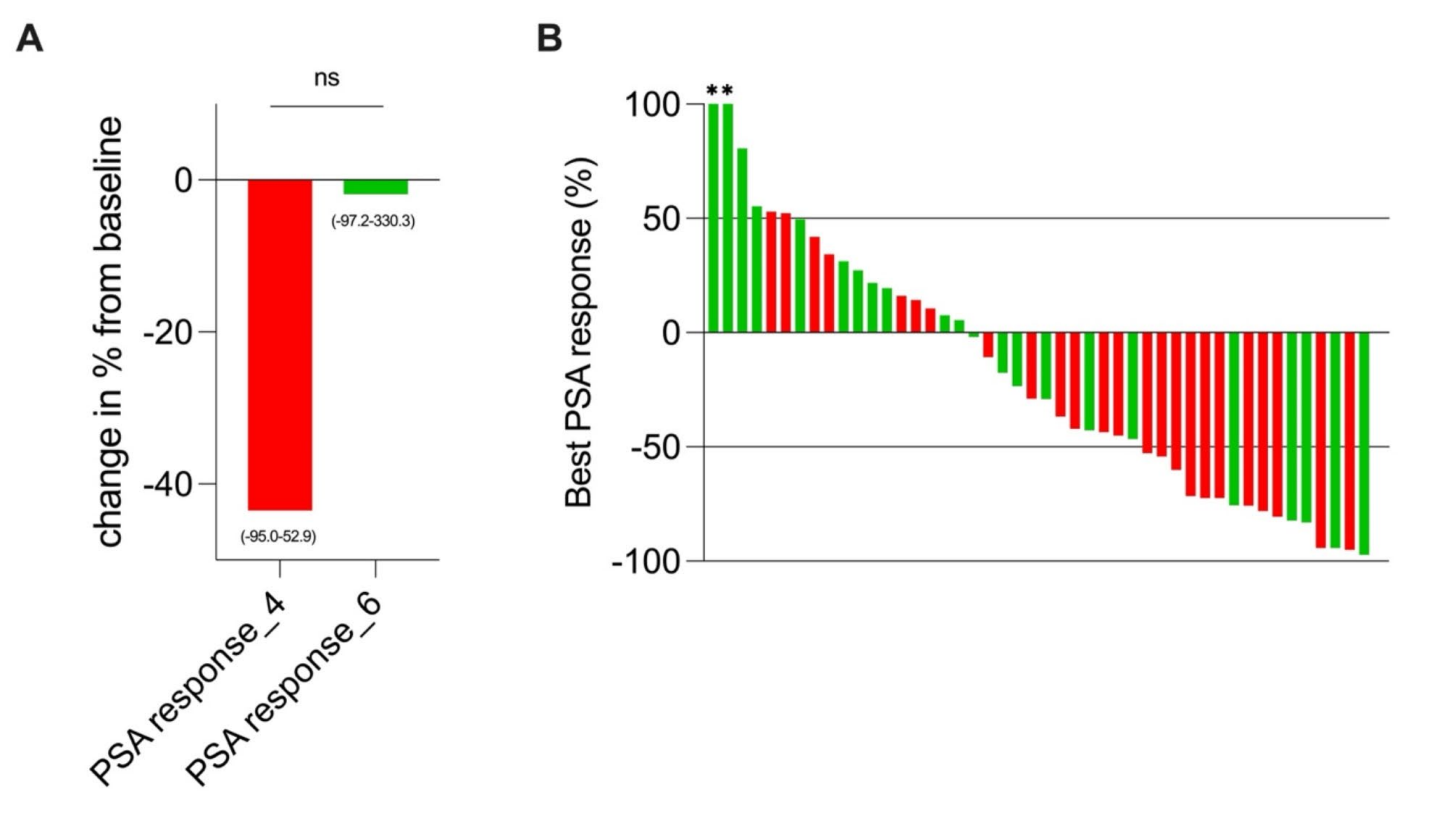
Best PSA response as relative changes from baseline. Best PSA response in patients treated every 4 weeks (red) compared to those treated every 6 weeks (green). Data are represented as median values, with their ranges in brackets. Waterfall plot showing response to treatment as measured by serum PSA. Best PSA response, defined as the smallest increase or greatest decrease in PSA from baseline in patients with 4-week treatment interval (red) and 6-week treatment interval (green). Asterics indicate patients with an increase of > 100% as the best PSA response

The median (range) early PSA response was − 28.5% (-97.2 to 217.9) after the first 3 cycles in the 4-week treatment interval group and 39.7% (-94.2 to 908.4) after the first 2 cycles in the 6-week treatment interval group. Additionally, at this time point, 14 out of 23 patients (60.9%) in the 4-week group and 9 out of 23 patients (39.1%) in the 6-week group experienced a PSA decline. Figure 4 presents best PSA response as a relative change from baseline and a waterfall plot illustrating best PSA response in patients receiving treatment at 4-week and 6-week intervals. In the 4-week treatment interval group, patients presented with a median PSA decline of -43.5% (range − 95.0-52.9; median PSA from 144.0 ng/ml (range 3.7–2760.0) to 79.6 ng/ml (range 1.56–1438.0), *p* = 0.052). Patients in the 6-week treatment interval group presented with a median PSA decline of -1.9% (range − 97.2-330.3; median PSA from 60.8 ng/ml (range 6.81–857.0) to 51.2 ng/ml (range 1.91–257.6), *p* = 0.8). The difference in best PSA response between both groups was not statistically significant (*p* = 0.2). A PSA decline ≥ 50% was achieved in 47.8% (*n* = 11) and 21.7% (*n* = 5) of patients in the 4-week and 6-week treatment interval groups (*p* = 0.12), respectively.

### Treatment outcome

**Fig. 5:**
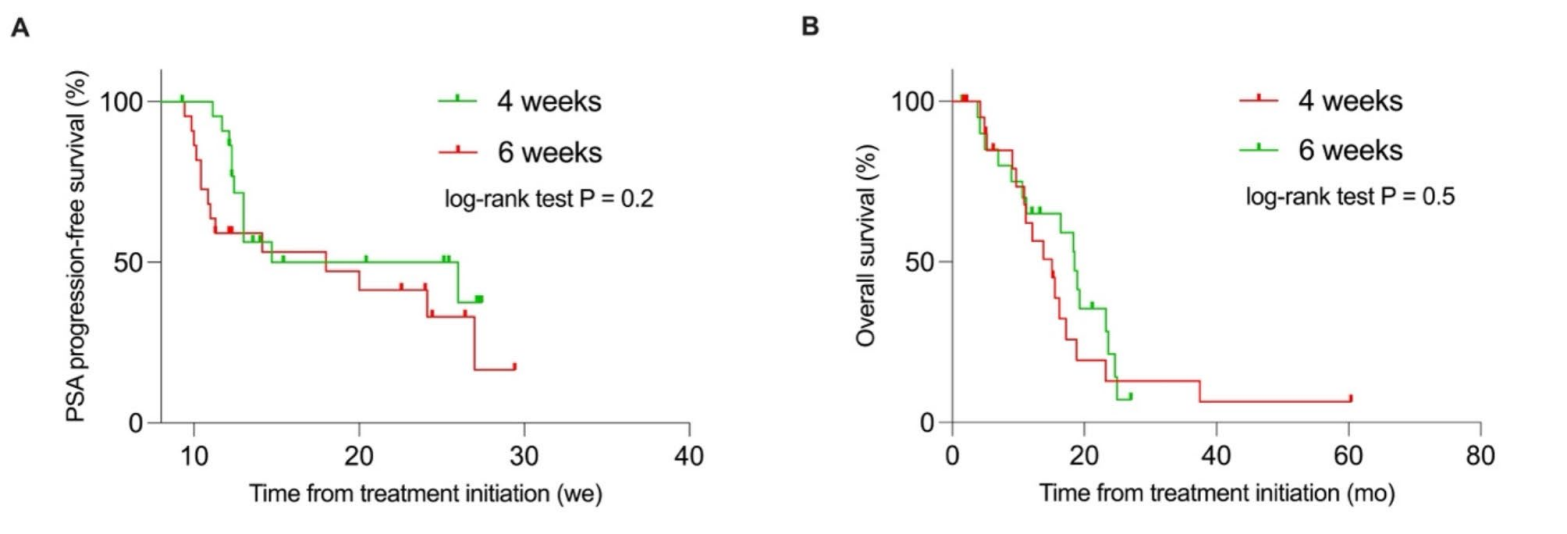
Kaplan-Meier survival curves. PSA-progresion-free survival and overall survival stratified by patients receiving treatment every 4 (red) and 6 weeks (green)

Median PSA-PFS tended to be longer in patients treated every 4 weeks compared to those treated every 6 weeks, although being not statistically significant (median PSA-PFS 26.0 weeks vs. 18.0 weeks; HR 0.6, 95% CI, 0.3–1.3; *p* = 0.2; see also Fig. 5). Although not statistically significant, OS in patients with a 4-week treatment interval tended to be shorter compared to those receiving treatment every 6 weeks (medians: 15.1 months vs. 18.4 months; HR 1.3, 95% CI, 0.6–2.7; *p* = 0.5; Fig. 5).

## Discussion

The results of our matched-pair analysis in mCRPC patients receiving consecutive cycles of ^177^Lu-labeled PSMA-RLT indicate a trend towards better treatment response and extended PSA-PFS in patients treated at 4-week compared to 6-week intervals, although not reaching statistical significance. However, OS was not significantly impacted by the different treatment approaches. Besides, patients with a 4-week treatment interval presented with a significant reduction in leukocyte and platelet counts, in comparison to those treated every 6 weeks, while both groups experienced only modest adverse events.

The assessment of treatment toxicity in the 4-week treatment interval group revealed a greater reduction in haemoglobin (13.9%), leukocytes (38.5%), and platelets (26.7%) compared to those treated in 6-week intervals with a decline of 7.8%, 18.2% and 10.7%, respectively. Significant differences were noted in the reduction of leukocytes (*p* = 0.047) and platelets (*p* = 0.02). This might be explained by the higher cumulative treatment activity in the 4-week treatment interval cohort (4-week: median 23.6 GBq, range 21.3–46.2 vs. 6-week: median 15.8 GBq, range 13.6–33.4, *p* < 0.0001) possibly leading to a higher bone marrow affection. Our results are in line with a recently published analysis investigating a 4-week treatment protocol in mCRPC patients reporting significant decreases in leucocyte and thrombocyte counts after three cycles of ^177^Lu-labeled PSMA-RLT with a mean activity of 7.315 GBq [8].

In our analysis, there were no relevant changes in creatinine levels between both groups. A potential explanation for this could be that the nephrotoxic effects of ^177^Lu-labeled PSMA-RLT may manifest later in patients [12]. However, in both patient groups, severe adverse events were relatively modest. Regarding severe anaemia, two grade III cases were observed in the 4-week treatment interval group (one with grade I and one with grade II at baseline) and one in the 6-week treatment interval group (already with grade III at baseline). Besides, more than 80% of patients in both groups already presented with a mild to moderate anaemia (Grade I/II) at baseline. Similar observations were made for leucocyte and platelet counts where grade III leucopenia occurred in one patient (Grade II at baseline) in the 4-week treatment interval group and thrombocytopenia of grade III was assessed in one patient (Grade II at baseline) in the 6-week treatment interval group. Both previously published analyses investigating 4-week treatment protocols reported comparable results with the appearance of only two grad III leukocytopenias and one grade III anaemia in a total of 45 mCPRC patients after three treatment cycles [8] and only two grade III leucopenias and one grade III thrombocytopenia in a total of 62 mCRPC patients receiving up to seven cycles of ^177^Lu-labeled PSMA-RLT [9]. Altogether, treatment with a shorter-intervalled regimen was generally well-tolerated potentially suggesting that the bone marrow deterioration is not primarily driven by the ^177^Lu-labeled PSMA-RLT. Moreover, it is more likely associated with a more advanced disease stage, as indicated by the prior treatments, including chemotherapy and hormonal therapy [13].

The early PSA response in the 4-week treatment interval group (median: -28.5%) was better compared to the 6-week treatment interval group (median: 39.7%). This was consistent with the results from the best PSA response, which favored patients with 4-week treatment intervals (median: -43.5%) over those with 6-week treatment intervals (median: -1.9%; *p* = 0.052). Additionally, noticeably higher rates of PSA declines ≥ 50% were achieved in patients in the 4-week treatment interval group compared to those receiving treatment every 6 weeks (47.8% vs. 21.7%; *p* = 0.12). These results suggest a dose-response relationship in treatment outcome, with higher cumulative treatment activities leading to better treatment responses. In line with this, patients with 4-week treatment intervals demonstrated a slightly extended PSA-PFS compared to those with 6-week treatment protocols (medians 26.0 weeks vs. 18.0 weeks, *p* = 0.2). PSA-PFS in our analysis was in accordance with previously reported median PSA-PFS of 0.4 years [8] and 25 weeks [9] in patients receiving ^177^Lu-labeled PSMA-RLT every 4 weeks, respectively. Furthermore, our results align with a recently published analysis reporting improved PSA response rates when treatment is applied in shorter intervals with 53.7% compared to 35.1% in patients receiving standard regimens of 7.5 GBq every 6 weeks versus 6.0 GBq every 8 weeks (*p* = 0.065) [7]. Thus, our results might suggest that a further intensified protocol, achieved by shortening the treatment intervals, could potentially lead to an improved treatment response and delayed PSA progression, indicating a possible buffer for additional improvement. Interestingly, our results are contradictory to a recently published analysis showing a slightly longer OS (12.7 vs. 11.3 months, *p* = 0.384) in patients treated with higher activities and shorter intervals, despite the lack of statistical significance [7]. In our study, OS was shorter in patients receiving treatment every 4 weeks compared to those on a 6-week regimen (medians 15.1 months vs. 18.4 months, *p* = 0.5). This might indicate that although patients seem to benefit in terms of treatment response and PSA-PFS, a further intensified treatment protocol does not necessarily lead to a significantly improved outcome. However, it must be noted that a median OS of 15.1 months in our 4-week treatment interval group is in line with recently reported data from the prospective phase III VISION trial [5]. There, a median OS of 15.3 months was reported for patients receiving ^177^Lu-labeled PSMA-RLT [5] suggesting that patients in the 6-week treatment interval group potentially represent a subgroup with exceptionally improved OS. Differences in OS between both groups might also be due to a potential selection bias, with patients in the 4-week treatment interval group representing a subgroup chosen by the treating nuclear medicine physician for critical treatment considerations such as rapid clinical decline, imaging and/or biochemical progression. Although an elaborate matching process was developed to minimize potential selection bias in the 4-week treatment group, differences in OS suggest that some bias cannot be completely ruled out. Supporting this hypothesis, our analysis revealed a significant difference in baseline PSA levels between the patient cohorts (4-week group: 144.0 ng/ml vs. 6-week group: 60.8 ng/ml; *p* = 0.006). Higher baseline PSA often indicates greater tumor burden and potentially poorer outcomes, although its prognostic value remains controversial. While some studies have shown that baseline PSA is significant for predicting treatment outcome [14, 15], this significance often disappears in multivariable analyses [15]. In line with this, other studies have found no link between baseline PSA and treatment response or outcome [16, 17]. However, considering the significant differences in baseline PSA, which were intentionally not included in the matching criteria due to large individual variances and its ambiguous impact on response and outcome, as well as the shorter OS observed in the 4-week group compared to the 6-week group, it cannot be ruled out that some selection bias may still be present. This is a limitation of the current study. Further limitations include the retrospective nature of this analysis. A major limitation of our analysis is that despite its match-pair design general differences in tumor biology cannot be excluded and differences might be biased by the small sample size. Another limitation is the variability in treatment intervals in both groups, based on the delays in the first interim PSMA-ligand PET/CT. Moreover, our analysis only assessed the impact of a 4-week treatment interval on toxicity, treatment response and outcome up to second interim PSMA-ligand PET/CT as some patients partly switched to a 6-week treatment interval after this.

## Conclusion

In conclusion, the results of our matched-pair analysis have indicated that the intensification of treatment intervals to 4 weeks, compared to the commonly practiced 6-week treatment intervals, can lead to improvements in treatment response and PSA-PFS in mCPRC patients receiving ^177^Lu-labeled PSMA-RLT with a standard activity of 7.4 GBq. However, it must be noted that such intensification of therapy did not lead to a substantial benefit in OS. Furthermore, the improvements in treatment response and PSA-PFS were compensated by a significantly greater impairment of bone marrow reserve, although major adverse events were only modest in both groups.

## Data Availability

The datasets supporting the conclusions of this study can be made available on reasonable request.

## Abbreviations

CTCAE v. 5.0: Common Terminology Criteria for Adverse Events version 5.0
CT: Computed tomography
CI: Confidence intervals
HR: Hazard ratio
LDH: Lactate dehydrogenase
mCRPC: Metastatic castration-resistant prostate cancer
miTNM: Molecular imaging TNM
OS: Overall survival
PET: Positron emission tomography
PSA: Prostate specific antigen
PFS: Progression-free survival
PSMA: Prostate specific membrane antigen
RLT: Radioligand therapy
